# Mitochondrial Roles and Cytoprotection in Chronic Liver Injury

**DOI:** 10.1155/2012/387626

**Published:** 2012-06-15

**Authors:** Davide Degli Esposti, Jocelyne Hamelin, Nelly Bosselut, Raphaël Saffroy, Mylène Sebagh, Alban Pommier, Cécile Martel, Antoinette Lemoine

**Affiliations:** ^1^AP-HP, Hôpital Paul Brousse, Service de Biochimie et Biologie Moléculaire, 14 Avenue Paul Vaillant Couturier, 94804 Villejuif Cedex, France; ^2^Inserm U1004, Université Paris 11, Institut André Lwoff, PRES Universud-Paris, Institut du Foie/Liver Institute, 14 Avenue Paul Vaillant Couturier, 94804 Villejuif, France; ^3^AP-HP, Inserm U785, Hôpital Paul Brousse, Service d'Anatomie Pathologique, 14 Avenue Paul Vaillant Couturier, 94804 Villejuif Cedex, France; ^4^Université Paris Sud 11, Faculté de Pharmacie, 5 rue Jean-Baptiste Clément, 92296 Châtenay-Malabry Cedex, France

## Abstract

The liver is one of the richest organs in terms of number and density of mitochondria. Most chronic liver diseases are associated with the accumulation of damaged mitochondria. Hepatic mitochondria have unique features compared to other organs' mitochondria, since they are the hub that integrates hepatic metabolism of carbohydrates, lipids and proteins. Mitochondria are also essential in hepatocyte survival as mediator of apoptosis and necrosis. Hepatocytes have developed different mechanisms to keep mitochondrial integrity or to prevent the effects of mitochondrial lesions, in particular regulating organelle biogenesis and degradation. In this paper, we will focus on the role of mitochondria in liver physiology, such as hepatic metabolism, reactive oxygen species homeostasis and cell survival. We will also focus on chronic liver pathologies, especially those linked to alcohol, virus, drugs or metabolic syndrome and we will discuss how mitochondria could provide a promising therapeutic target in these contexts.

## 1. Introduction

Mitochondria are intracellular double membrane-bound structures that provide energy (ATP) for intracellular metabolism. The intramitochondrial metabolism includes Krebs cycle and beta-oxidation. Mitochondria are also essential for assembly of iron sulfur clusters and regulation of calcium homeostasis. However, mitochondria are not only the cell's powerhouse, organelles whose particular architecture and biochemical composition enable the maximization of energy production by oxidative phosphorylation (OXPHOS), but they also have a second crucial function, namely, the control of cell death following activation of intracellular signaling cascades or death receptor-mediated pathways [1]. Indeed, the mitochondrial membrane permeabilization (MMP) is the decisive event that marks the transition from survival to death. Thus, mitochondrial membranes integrate proapoptotic and antiapoptotic signals coming from microenvironment or from other intracellular organelles, such as endoplasmic reticulum or lysosomes, defining the ultimate cell fate [1, 2]. The number and functions of mitochondria can vary depending on age, sex, organ, and physiological or pathological conditions that are still unknown [3–5].

Mitochondrial dysfunctions are frequently described as early and initiating events in various chronic pathological conditions in different tissues and organs, such as liver, brain, or heart [6–8]. Most forms of chronic liver diseases are associated with the accumulation of damaged mitochondria responsible for abnormal reactive oxygen species (ROS) formation, glutathione (GSH) depletion, protein alkylation, and respiratory complex alterations. Depending on their nature and severity, the mitochondrial alterations may induce lipid accumulation, apoptosis, and/or necrosis leading to hepatic cytolysis and inflammation. These pathological events can correspond to different clinical features, such as lactacidosis, hypoglycemia, elevated serum transaminases, higher conjugated bilirubinemia, and hyperammonemia. However, a growing body of literature has also shown that demised cells with damaged mitochondria can develop cytoprotective mechanisms to ensure cellular energy homeostasis and limit cell death [9–12]. These mechanisms consist in both activation of intracellular pathways targeting mitochondria function and intercellular and interorgan signaling to coordinate adaptive metabolic responses within the organism as a whole. The regulation of the mitochondrial biogenesis and/or turnover (by general autophagy or specific mitochondria-targeted mitophagy) plays an important role in the balance of cell survival and cell death [13]. This balance is importantly linked to the energy metabolism homeostasis, in particular with ATP synthesis, as it has been reported in some chronic liver pathologies, such as steatosis and nonalcoholic steatohepatitis (NASH) [14].

In this paper, we will focus on the role of mitochondria in liver physiology and pathologies, especially those linked to alcohol, virus, drugs, or metabolic syndrome and we will discuss how mitochondria could provide a promising therapeutic target in these contexts.

## 2. Mitochondria in Liver Physiology

The liver is one of the richest organs in terms of number and density of mitochondria. The density of mitochondria is different in various tissues depending upon numerous factors, mostly the demands of oxidative phosphorylation. A study showed that in nontumorous liver tissue the copy number of mitochondrial DNA (mtDNA) in male patients affected by hepatocellular carcinoma (HCC) was lower than that of the female patients (5308 ± 484 versus 8027 ± 969, *P* < 0.05) [4]. Since each mitochondrion can host from two to ten copies of mtDNA [5], we can assume that in the liver, the number of mitochondria could range from 500 to 4000 per hepatocyte.

In this chapter, we will review the role of mitochondria in hepatic metabolism, reactive oxygen species (ROS) homeostasis, and cell death regulation.

### 2.1. Mitochondria Are Essential in the Hepatic Metabolism

The liver is an essential life organ in all mammals and plays a central role in the homeostasis of carbohydrate, lipid, and protein metabolism of the organism. The liver is a main target of insulin and glucagon signaling and contributes to balancing glucose blood levels by regulating glycogen synthesis and gluconeogenesis in hepatocytes [15]. It is also a key organ in maintaining lipid homeostasis: it is the main site of fatty acid oxidation together with the muscle (mainly *β*-oxidation taking place into the mitochondria) and it is the sole organ able to synthesize fatty acids by *de novo *lipogenesis [16]. Finally, the liver is a key regulator of protein metabolism for the entire organism as hepatocytes synthesize essential proteins such as albumin and lipoproteins and allow ammonia detoxification through the urea cycle [17].

**Figure 1:**
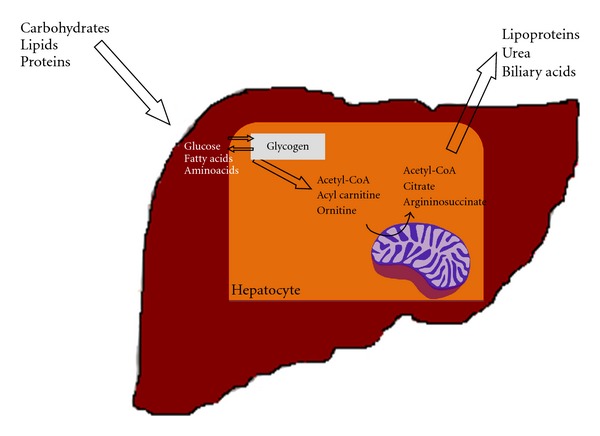
The role of hepatocyte mitochondria in liver metabolism. The liver is a central organ for the homeostasis of carbohydrates, lipids and proteins metabolism. In this context, hepatocyte mitochondria are essential in regulating the flux of metabolites in the cell in order to adjust energetic demand, ammonia detoxification, or anabolic pathways. Energy demand is met by complete oxidation of acetyl groups coming from glycolysis through tricarboxyilic acid cycle or of acyl groups coming from lipolysis through *β*-oxidation. Moreover, mitochondria are a unique site for metabolizing ammonia into the less toxic urea. Then, mitochondria provide shuttle proteins that allow specific addressing to anabolic pathways, as in the case of citrate transport protein (CTP) (see text for details).

In this context, the mitochondria provide the hub that integrates these pathways, serving as a critical site for the production and exchange of metabolic intermediates (Figure 1). It plays a critical role in orchestrating these complex metabolic networks in order to maintain proper homeostasis.

Mitochondria are largely involved in glucose metabolism, as the pyruvate dehydrogenase (PDH) complex is expressed in the mitochondrial matrix. It is composed by 5 subunits (pyruvate dehydrogenase, E1 alpha and E1 beta; dihydrolipoamide S-acetyl transferase, E2; and lipoamide dehydrogenase, E3 and E3BP). It catalyzes the conversion of pyruvate, the last metabolite of aerobic glycolysis, to Acetyl-CoA and CO_2_. In the last ten years evidence has accumulated showing an important involvement of liver mitochondria in insulin resistance. In insulin resistant states, alterations in mitochondrial function, structure, and organization have been described [18]. In particular, a decrease in respiration and ATP production has been frequently described and the decreased efficiency is often attributed to excessive mitochondrial ROS production inducing respiratory chain protein oxidation [14, 18, 19].

Concerning lipid metabolism, few mitochondrial proteins play key roles in catabolism as well as in anabolism. The carnitine palmityl transferases I and II (CPT I-II) are expressed at the mitochondrial outer membrane (MOM) and mitochondrial inner membrane (MIM), respectively, and are essential for acyl-CoA transport and subsequent fatty acid *β*-oxidation in liver and muscle. A mitochondrial transport protein, the citrate transport protein (CTP), allows acetyl-CoA to be transported from mitochondria to the cytosol in the form of citrate in order to be used as building block in hepatic *de novo *lipogenesis [20]. Hepatic mitochondria are essential also in protein metabolism. Nitrogen enters the liver as free ammonia and amino acids, mostly glutamine and alanine [17]. Enzymes involved in ammonia detoxification and urea synthesis (glutamate dehydrogenase, carbamoyl phosphate synthetase I and ornithine transcarbamylase) are exclusively expressed in the hepatocyte mitochondria. Indeed, the first step in the urea cycle for ammonia detoxification and disposal is located at mitochondria and mediated by the enzyme carbamoyl phosphate synthetase 1 (CPSI). CPSI is allosterically regulated by cytosolic N-acetyl-L-glutamate (NAG) [21]. Ammonia can be also converted to glutamine by the glutamine synthetase (GS) catalyzing the condensation of glutamate and ammonia and, *vice versa*, ammonia can be generated by glutaminase. Therefore, an increase in blood ammonia depends on the activity of the enzyme glutamine synthetase, the glutamine/glutamate cycle, and the tissue capacity to eliminate toxic ammonia. Mitochondria represent a major site of glutamine metabolism, as both glutaminase and GS are mitochondrial processes in the liver. Interestingly, in absence of glucose but with high glutamine concentrations, mitochondrial structure and dynamics change towards a more condensed configuration and extended reticulum [22]. Moreover, urea and glutamine metabolism are differently distributed in the hepatic acinus. Ammonia is taken up by periportal hepatocytes, metabolized to urea via the urea cycle and excreted through the kidneys. Any ammonia escaping detoxification is usually trapped by perivenous hepatocytes, where it is converted to glutamine via glutamine synthetase [23]. Indeed, urea synthesis enzymes and glutaminase are expressed in periportal hepatocytes, while glutamine synthetase is expressed in perivenous hepatocytes [17]. Then, the periportal region has a low affinity but a high capacity for ammonia detoxification. Hepatic GS allows ammonia scavenging, and when liver impairment is present, a diminished detoxification capacity is observed. GS has a short half-life and its activity is regulated and modulated by several mediators and hormones. The brain also uses glutamine synthesis for metabolizing ammonia and for deamination in the presynaptic terminals to produce glutamate, an important excitatory neurotransmitter. When it accumulates, it is taken up by the astrocytes and recycled back to glutamine, the “storage centre” for free ammonia [24, 25]. It is interesting to note that the different subcellular localization of GS (mitochondrial in hepatocytes and cytoplasmic in astrocytes) has been considered a partial explanation to the higher toxicity of ammonia in the brain than in the liver [26]. However, the exact role of mitochondrial dysfunctions in hyperammonemia still needs to be addressed, in particular for chronic liver disease. However, liver and mitochondria metabolism are directly involved in the homeostatic balance of brain ammonia, glutamine, and glutamate.

### 2.2. Mitochondria Are Essential in Reactive Oxygen Species Homeostasis

**Figure 2:**
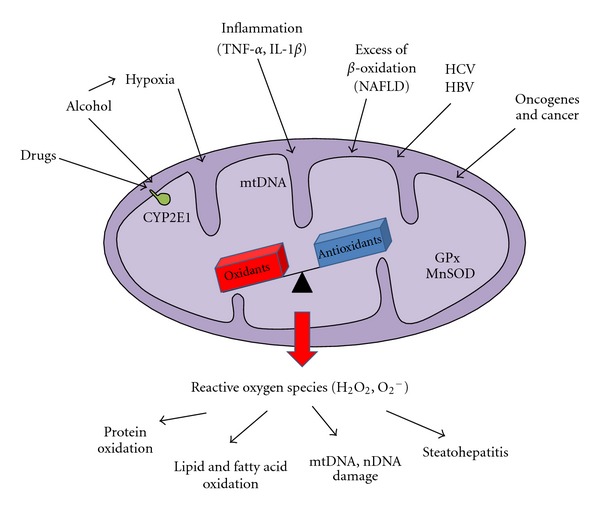
The role of hepatocyte mitochondria in reactive oxygen species homeostasis. Mitochondria are a physiological source of reactive oxygen species (ROS). In this context, ROS exert a signaling role in cell proliferation and differentiation. However, different types of stress can target directly or indirectly hepatocyte mitochondria, such as drugs, virus, hypoxia, inflammatory cytokines, excess of *β*-oxidation, ectopic expression of cytochromes P450. In this case, overproduction of ROS may damage both mitochondrial and other cellular components, such as OXPHOS protein subunits, lipid membranes, mitochondrial, or nuclear DNA. These cellular lesions can favor the development of tissue lesions, such as steatohepatitis or hepatocellular carcinoma.

Mitochondria are the intracellular organelles devolved to energy (ATP) production in all eukaryotic cells through oxidative phosphorylation (OXPHOS). OXPHOS is allowed by the four multiprotein complexes of the mitochondrial respiratory chain (MRC) and by the ATP synthase. OXPHOS physiologically produces reactive oxygen species (ROS) and *in vitro* estimations lead to considering that up to 2% of oxygen consumption results in superoxide anion generation [27]. Thus, mitochondria are a main source of ROS (Figure 2). ROS are produced during oxidative metabolism mainly by the complexes I, III, or IV of the electron transport chain, where electrons can prematurely reduce oxygen, resulting in the formation of superoxide radical [27–30]. In the normal state, most of the ROS generated by the MRC are detoxified by the mitochondrial antioxidant enzymes, such as SOD2/MnSOD, which convert superoxide to hydrogen peroxide, subsequently detoxified by GSH peroxidase. The remaining nondetoxified ROS diffuse out of mitochondria and serve as signaling molecules vital for normal cellular functions [31]. These physiological ROS are involved in specific cellular pathway aimed to adapt global metabolism to transient or chronic stress conditions. It is interesting to note that ATP synthase may also have a regulating role in ROS production. Actually, in the experimental model of aging provided by the fungus Podospora anserine, characterized by mitochondrial etiology of aging, the alpha subunit of ATP synthase functions as a sensor of oxidative stress and provides an intramolecular quencher (at the residue Trp503) for ROS [32]. Moreover, a recent mechanism that seems to buffer ROS excess has been described in physiological and pathological conditions. The expression of uncoupling proteins (UPCs) promotes a controlled uncoupling of proton flux from the ATP synthase and could lead to decreased ROS production [33].

### 2.3. Mitochondria Are Essential in Cell Survival

Mitochondria are the essential actor in keeping the balance between cell survival and cell death, in particular in hepatocytes, where they trigger the intrinsic pathway of apoptosis and are also involved in necrotic cell death. The regulation of membrane permeability is the main mechanism that makes the cells shift from survival to cell death. The MOM is permeable to solutes of molecular mass (MM) ≈ 6 kDa due to the presence of channels, such as the voltage-dependent anion channel (VDAC), which belongs to the porin subfamily. However, with an estimated pore diameter about 2.6–3 nm, VDAC would not allow the passage of a folded protein like cytochrome c. In contrast, the MIM is almost totally impermeable and transport of ions and solutes is granted by mitochondrial carrier proteins. Most mitochondrial proteins exhibit dual functions, a vital metabolic function, and a lethal pro-apoptotic function. This applies to various channels: voltage-dependent anion channel (VDAC), adenine nucleotide translocase (ANT), Bax, *t*-Bid, Bak; receptors (e.g., TOM22); chaperones (cyclophilin D, CypD), as well as oxidoreductases (apoptosis-inducing factor, AIF).

During apoptosis, many signals can converge to the mitochondrion to MMP, the rate-limiting step in the execution of the death process [1]. MMP is regulated mainly by the members of Bcl-2 family, members of the PTP complex (VDAC, ANT, CypD) and lipids [1]. Bcl-2 family is composed of pro-apoptotic proteins (e.g., Bax, Bak, Bid, Bik, Bnip3) and anti-apoptotic members (Bcl-2, Bcl-x_L_, Bcl-w, Mcl-1, A1). Pro-apoptotic proteins favor MMP by translocating to MOM and forming mega channels, mainly by oligomerization (e.g., Bax-Bak oligomers or Bax-VDAC complexes), while anti-apoptotic members stabilize MOM and tend to prevent MMP [1, 34–36]. Accumulation of modified lipids (e.g., oxidized cardiolipin, ceramide) and ions (e.g., Ca^2+^) in the mitochondrion can also influence MMP [37]. Moreover, the intracellular milieu, such as pH, ROS, and ATP levels can contribute to define a permissive environment for MMP execution [1]. Multiple mechanisms can mediate MMP, depending on the cell type and the death stimuli. They can affect either the MOM, or both mitochondrial membranes (MOM+MIM). In the MOM model, intermembrane space proteins are released into the cytosol by passage through large proteic/lipidic channels while, in the MOM+MIM model, intermembrane space proteins are freely released into the cytosol through the MOM ruptures. Nevertheless, these two models can coexist in conditions involving on the one hand the translocation of the truncated form of Bid (tBid) to mitochondria, and in the other hand mitochondrial Ca^2+^ accumulation and ROS increase, as observed in conditions of endoplasmic reticulum stress [1]. In the MOM+MIM model, the contribution of the permeability transition pore (PTP) seems to play an important role. The PTP consists of a multiprotein complex (PTPC) and various proteins are involved in its opening. Long lasting opening of PTPC increases MIM permeability and, in the presence of adequate amounts of ATP, would lead to apoptotic cell death [1]. PTPC opening is highly sensitive to Ca^2+^, prooxidant agents, pro-apoptotic Bcl-2 family members and some chemotherapeutics agents [38]. However, Ca^2+^-induced PTP opening has been also reported to induce necrotic cell death, in particular when intracellular ATP levels are too low to allow apoptosis execution [39].

Once initiated, MMP leads to the release into the cytosol of caspase-dependent proteins (i.e., cytochrome c or Smac/DIABLO) and caspase-independent proteins (such as apoptosis-inducing factor, AIF, or EndoG) with consequent coordinated cell degradation [40]. Concomitantly, MMP provokes a mitochondrial failure with dissipation of the inner membrane potential (ΔΨ*m*), subsequent arrest of OXPHOS and ATP synthesis, and increased ROS level. Therefore, MMP constitutes a point of no return of the activation cascade of cell death [41].

## 3. Mitochondria in Liver Pathology

Most liver pathologies, including alcoholic liver disease, nonalcoholic fatty liver disease (NAFLD) and nonalcoholic steatohepatitis (NASH), drug-induced hepatotoxicity, viral hepatitis, and HCC, are characterized by mitochondrial dysfunctions. Moreover, during liver surgery, liver cells, in particular hepatocytes and endothelial cells suffer ischemia/reperfusion (I/R) injury. In the liver, as well as in other organs such as brain and heart, I/R injury involved mitochondrial permeability transition [1]. Since these abnormalities affect all the aforementioned physiological functions of mitochondria, we will review their roles in liver pathologies with a particular focus on the aspects of cell death regulation, alteration of hepatocyte metabolism, and disruption of ROS homeostasis.

### 3.1. Mitochondria in Cell Death Regulation

**Figure 3:**
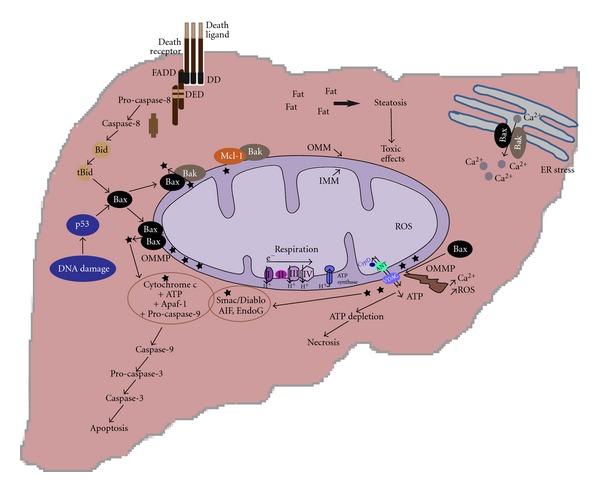
The mitochondria are central organelles in determining cell fate in liver diseases. Hepatocyte cell death is common to many liver diseases. Different stress stimuli can induce death signaling, such as toxic free fatty acids, DNA damage, endoplasmic reticulum (ER) stress observed in metabolic disease. In these contexts, mitochondria are essential to determine cell fate, as in hepatocyte the activation of the intrinsic pathway of apoptosis by cell death receptors is not usually sufficient to induce cell death and liberation of proapoptotic factors from mitochondria is a mostly necessary event. Moreover, previous alterations of mitochondrial function causing decreased ATP synthesis can induce a shift from apoptotic to necrotic cell death.

Mitochondria are key organelles in the development of liver diseases characterized by hepatocyte death and subsequent inflammation (Figure 3). Actually, increased hepatocyte apoptosis has been correlated with inflammation, fibrosis, and cell turnover, conditions that are permissive for the development of HCC [2]. Hepatocyte mitochondria are essential in making effective the extrinsic pathway activated by many ligands, such as Fas, TRAIL or TNF-*α* [2]. Moreover, constitutive expression of both anti-apoptotic proteins Bcl-x_L_ and Mcl-1, belonging to the Bcl-2 family, is required to avoid spontaneous caspase 3/7 activation, suggesting essential cytoprotective functions of these proteins in the hepatocyte [42, 43]. Bcl-2 is not constitutively expressed in the liver; however, it can be induced in order to cope with I/R, as shown in ischemic preconditioning during partial hepatectomy [44, 45].

Fas- and TRAIL-mediated apoptosis are involved in viral hepatitis, playing a crucial role in the elimination of infected cells and the hepatitis viral core protein binds Mcl-1 impairing its cytoprotective function [46–48]. TNF-*α* is secreted by infiltrating cytotoxic T lymphocytes during HBV infection and its apoptotic effect seems to be mediated by HBVx protein [2]. Mitochondrial apoptosis is also involved in the pathogenesis of NAFLD and in NASH [15]. In an experimental model using mice fed with a methionine and choline deficient diet, apoptosis was induced by an increase hepatic expression of functional p53, with a concomitant increase in the cleavage of Bid to tBid and a decrease expression of Bcl-x_L_ [49]. Moreover, p53 was also responsible for TRAIL receptor expression, linking intrinsic and extrinsic apoptosis pathway in NASH [49]. Recently, saturated free fatty acids have been shown to activate the proapoptotic proteins Bim and Bax via JNK, thus inducing MMP, and also increase ROS production [50].

Hepatocyte necrosis is usually considered an accidental (nonprogrammed) form of cell death, resulting from metabolic failure and consequent rapid ATP depletion [51]. It has been firstly described during I/R injury following liver transplantation or hepatectomy, but it is also described in NASH. In fact, hepatocytes necrosis is associated with significant inflammatory response, due to the liberation of IL1-*β*, TNF-*α* and other newly described proinflammatory proteins, namely, damage-associated molecular-pattern (DAMP) molecules, such as HMGB1, that activate innate immunity response, such liver resident macrophages (Kupffer cells) and polymorphonuclear cells [45, 51–54]. Recently, accumulated evidence indicates that necrosis can also occur in a regulated manner and that the liberation of cytokines from dying cells can function as a sentinel signal alerting to the need for defensive response [51]. This regulated or programmed necrosis (necroptosis) is initiated by death receptors, like apoptosis, but requires activation of specific kinases (receptor interacting proteins 1 and 3) and its execution involves the active disintegration of mitochondrial, lysosomal, and plasma membranes [55]. Interestingly, in the context of I/R injury, the PTPC opening seems to be a common event anticipating both necrotic cell death and apoptosis, reinforcing the idea that programmed necrosis may be involved in clinical and pathological contexts. In an experimental model of orthotopic liver transplantation in rats, inhibition of PTP by cyclosporine A or acidic pH improved mitochondrial and hepatocellular functions, in particular decreasing the percentage of apoptotic cells but not of necrotic cells [56, 57]. These results seem to confirm the concept that apoptosis is typically an early event in hepatocyte injury. Importantly, in steatotic livers submitted to ischemia/reperfusion, necrosis is predominant compared with normal liver in which apoptosis is the main form of cell death [52, 53]. This difference has been partially linked to the metabolic/energetic difference between steatotic livers and normal liver [52] since fatty liver mitochondria have a decreased content of cytochromes c oxidase, produce superoxide anion and H_2_O_2_ at increased rate and have an increase content in UPC-2 compared with normal livers, resulting in decreased ATP production that affects apoptosis execution, and favors necrosis [58].

### 3.2. Mitochondria in Alteration of Hepatocyte Metabolism

The aforementioned data suggest that mitochondria may be the convergence point between various metabolic stresses and cell death in hepatocyte. In this context, it merits noting that the cytosolic glucokinase, or hexokinase IV, the hepatic/pancreatic isoform of hexokinase, has been recently reported to be associated to mitochondrial proteins, such as Bad, at the MOM [59, 60]. The association of the pro-apoptotic protein Bad with the glucokinase suggests that a close integration exists between the pathways of glucose metabolism and apoptosis [59].

Many studies on obese, diabetic, or NASH patients have shown functional and structural abnormalities in hepatocyte mitochondria, such as OXPHOS impairment or megamitochondria [61]. Interestingly, both increased or decreased *β*-oxidation in insulin resistant hepatocytes has been reported as characteristic of liver steatosis and insulin resistance [16, 62, 63]. Decrease in *β*-oxidation activity induces diacylglycerol (DAG) accumulation and steatosis in the hepatocyte with concurrent activation of PKC pathway and inhibition of insulin signaling [62]. In insulin-resistant patients an increased activity of hepatic *β*-oxidation was observed and this was correlated to an increase in ROS production [61, 64]. Elevated *β*-oxidation could be an adaptive mechanism to limit free fatty acid lipotoxicity, thus providing large amounts of reduced equivalents (NADH) regardless of energetic requirements finally promoting ROS production due to impairment of respiratory chain [18]. These results linked mitochondrial metabolic dysfunctions to oxidative stress due to increased ROS production.

### 3.3. Mitochondria in Disruption of ROS Homeostasis

Increased ROS production has been described in most liver pathologies. Augmented generation of mitochondrial ROS has been reported in various animal models of NASH, namely, genetically obese-diabetic ob/ob mice [58] and rats fed with a choline-deficient diet [65]. Moreover, mitochondria can be an ectopic site of cytochromes P450 2E1 expression [61, 66], where it can produce ROS and induce lipid peroxidation, as shown in the liver of an experimental model of diabetic rat [67].

Mitochondrial dysfunctions and ROS generation have been clearly shown in alcoholic liver disease [68]. Excessive ethanol consumption perturbs sinusoidal blood flow, inducing ischemia regions, and causes increased production of TNF-*α*, which induces inflammatory cell infiltration and an increase in hepatic O_2_ consumption [69, 70]. Chronic ethanol consumption induces profound disruption in mitochondrial metabolism, in particular decreasing the rate of ATP synthesis, thus placing hepatocytes under bioenergetic stress [68]. Under alcohol feeding, mitochondria contribute to the production of ROS in hepatocytes through various mechanisms. Ethanol metabolism increases the availability of NADH, resulting in a more reduced state of complexes I and III of the respiratory chain with a consequent increased probability of superoxide ion production [71]. Moreover, chronic alcohol consumption decreases mitochondrial protein synthesis mainly due to alcohol-mediated damage to mtDNA, contributing to decreased functioning of the oxidative phosphorylation system [72–74].

Mitochondrial ROS also play an important role in viral hepatitis. HCV core protein directly interacts with mitochondria and inhibits complex I activity, inducing an increased production of mitochondrial ROS, reducing threshold for Ca^2+^ and ROS-induced MMP [75]. Moreover, it has been recently shown that during HBV infection, HBx protein interacts with mitochondria, increasing ROS production [76]. The increase in ROS production was necessary, although insufficient, to induce the proinflammatory enzyme cyclooxygenase 2 (COX-2), linking mitochondrial dysfunction with liver inflammation in HBV infection [76]. Numerous investigations have shown that mitochondrial dysfunction is a major mechanism of drug- (or drug-metabolite-) induced liver injury [77]. Different mechanisms of mitochondrial dysfunction have been described in drug-induced hepatotoxicity, including membrane permeabilization, OXPHOS impairment, inhibition of fatty acid oxidation, and mtDNA depletion, and it appears that overproduction of reactive oxygen species by the damaged mitochondria could play a major role [77]. Finally, there is evidence showing a role of ROS in hepatocarcinogenesis [78]. Chemical hepatocarcinogens, such as the mycotoxin aflatoxin B1 and 2-acetylaminofluorene (2-AAF), induced increased ROS production in hepatocytes. In particular, 2-AAF altered mitochondrial redox cycling and it activated NADPH oxidase, an important ROS producing enzyme, through PI3K/Akt pathway [79–81]. Growth factors and activated oncogenes also induce ROS overproduction. Cultured cells treated with epidermal growth factor (EGF) and platelet-derived growth factor (PDGF) showed increased levels of H_2_O_2_ [82, 83]. Double transgenic mice bearing liver-targeted expression of transforming growth factor and the oncogene* c-myc* develop HCC as early as 4 and 8 months of age and elevated ROS levels associated with lipid peroxidation, mitochondrial damage and decreased GSH were already observed at 2-3 months of age [84].

Thus, it is clear that even a mild dysfunction of mitochondria in the liver could lead to hepatic and systemic pathological conditions and the identification of type and timing of mitochondrial lesions could allow major advancement in prevention, early diagnosis and treatment of systemic and liver diseases.

## 4. Mitochondria in the Cytoprotection of Damaged Liver Cells to Ensure Homeostasis in Chronic Liver Diseases

Mitochondrial dysfunction is described in various hepatic diseases or lesions, such as NAFLD, I/R injury, drug toxicity or hepatocellular carcinoma, and it is often detected as an early alteration, suggesting its causative effect [6, 85–87]. Cells have developed different mechanisms to keep mitochondrial integrity or to prevent the effects of mitochondrial lesions, such as disposal of damaged mitochondria by autophagy/mitophagy, increased biogenesis of mitochondria or regulation of signaling pathways to ensure energy metabolism and limit cell death and inflammatory response.

### 4.1. Increased Biogenesis of Mitochondria

**Figure 4:**
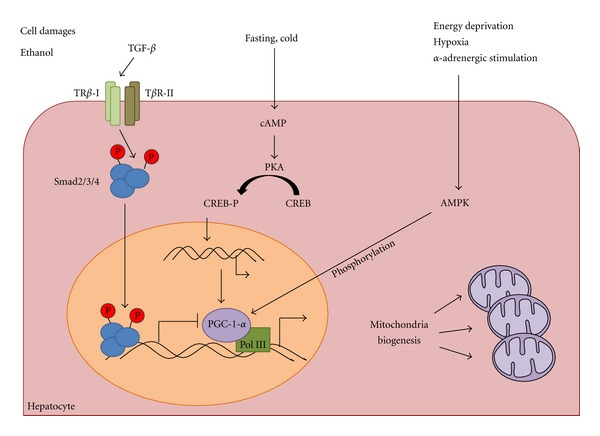
Mitochondria biogenesis allows tissue adaption under stress. Mitochondria biogenesis has been recently recognized as a central pathway in the adaptation of stress conditions in the liver, such as fasting, energy deprivation, hypoxia, or alcohol consumption. Different signaling pathways converge on the master regulator of mitochondria biogenesis, PGC1-alpha. In particular, AMPK and PKA signaling may activate gene transcription controlled by PGC1-alpha, while the TGF-*β* has been shown to inhibit PGC1-alpha-induced gene transcription.

Regulation of mitochondria biogenesis is one of the mechanisms developed by cells to keep mitochondrial integrity or to prevent the effects of mitochondrial lesions. The peroxisome proliferator-activated receptor gamma coactivator-1 alpha (PGC-1 alpha) belongs to the family of PGC-1 transcriptional coactivators (PGC-1 alpha, PGC-1 beta and PRC), which have been shown to be master regulators of mitochondrial biogenesis, and cellular energy metabolism in many organs, including liver [88, 89]. PGC-1 alpha is present at low but inducible levels in the liver where it also regulates most of the metabolic pathways, including gluconeogenesis, fatty acid *β*-oxidation, ketogenesis and heme biosynthesis (Figure 4) [90–93]. Under stress conditions, such as low temperature, fasting or energy deprivation, PGC-1 alpha is activated both transcriptionally by cAMP response element binding protein (CREB) and post-traductionally by AMP-activated-protein-kinase- (AMPK-) induced phosphorylation and SIRT1-mediated deacetylation [89]. Following PGC-1 alpha activation, different nuclear factors are subsequently activated. In particular, an activation of the nuclear respiratory factors 1 and 2 (NRF-1 and NRF-2) is observed and is followed by increased expression of multiple mitochondrial proteins. Moreover, PGC-1 alpha activates the nuclear receptors peroxisome proliferator-activated receptor alpha (PPAR alpha) and the estrogen-related receptor alpha (ERRalpha) both promoting the transcription of genes involved in *β*-oxidation, such as medium chain acyl-CoA dehydrogenase and carnitine palmitoyltransferase-1A (CPT-1A) [94, 95]. The absence of adequate levels of PGC-1 alpha is correlated with mice developing fasting hypoglycemia and hepatic steatosis, while mouse models of type 1 and type 2 diabetes showed high hepatic levels of PGC-1 alpha [90]. However, it has been recently shown that the different tissue-specific functions of PGC-1 alpha are tightly and independently regulated [96]. In particular, S6 kinase-1 (S6K1), activated in the liver upon feeding, can phosphorylate PGC-1 alpha, decreasing its capacity to turn on genes of gluconeogenesis, while keeping the functions of activator of mitochondrial and fatty acid oxidation genes intact [96]. S6K1, liver kinase B1 and AMPK are key kinases in the regulation of energy metabolism in the liver. Actually, AMPK is emerging as a kinase that links energy metabolism to mitochondrial function and biogenesis since components downstream of AMPK may contribute to stabilize mitochondrial membrane potential for hepatocyte survival, strengthening the relationship between fuel metabolism and cell survival [10]. Actually, in the liver, the activation of AMPK has been shown to decrease gluconeogenesis and fatty acid synthesis, to increase fatty acid oxidation and mitochondrial biogenesis and this could be linked to PGC-1 alpha phosphorylation, as previously observed in skeletal muscle [97, 98]. Interestingly, the hepatitis B virus (HBV) uses the transcriptional machinery involved in the hepatic response to fasting for its own amplification, thus HBV life cycle is under the control of PGC-1 alpha that could be a new target for antiviral therapy [99]. The dynamic changes in mitochondrial morphology, connectivity, and subcellular distribution are also major mechanisms in cellular homeostasis. They are critically dependent on a highly regulated fusion and fission machinery. Mitochondrial function, dynamics, and quality control are vital for the maintenance of tissue integrity [100]. In the liver, it has been shown that specific protection against hepatocyte mitochondrial dysfunction plays a preventive role in early stages of fibrogenesis, delaying, but not avoiding, its onset [101]. In this context, it is interesting to note that TGF-*β*/Smad3 signaling pathway, known to be implicated in liver fibrogenesis, has been shown to regulate glucose and energy homeostasis. Smad3-deficient mice are protected from diet-induced obesity and diabetes and Smad3 acts as a repressor of PGC-1*α* expression, thus suggesting a link between failure in mitochondrial biogenesis, metabolic syndrome, and liver fibrosis [102].

### 4.2. Autophagy and Mitophagy as Mechanisms to Limit Mitochondrial Lesions

Autophagy is a cellular pathway by which cytoplasmic materials, including organelles, reach lysosomes for degradation. Autophagy may occur either as a general phenomenon, for instance, during nutrient deprivation, or it can specifically target distinct cellular structures, such as damaged mitochondria (mitophagy) [13]. An important interplay exists between induction of autophagy and mitochondria. Actually mitochondria seem to have a key role in general autophagy as they may supply membranes for the biogenesis of autophagosomes during starvation [103]. Moreover, low ATP production or enhanced ROS generation by mitochondria induces general autophagy [104, 105]. The selective removal of mitochondria by mitophagy regulates mitochondrial number to match metabolic demand and is considered a form of quality control to remove damaged mitochondria [106]. Induction of general autophagy by a sublethal stress before a lethal stress can protect cells against cell death [13]. Indeed, we showed that ischemic preconditioning of livers previously treated by chemotherapy or steatotic livers induced autophagy and decreased necrosis without altering apoptosis. [45, 54]. The elimination of damaged mitochondria has been correlated to resistance of residual mitochondria to MMP and opening of PTP, two early events of apoptotic/necrotic cell death. This can be explained either by the removal of mitochondria that have a low threshold for permeabilization or by the fact that MMP or PTP opening occurs in a fraction of mitochondria and may activate autophagic disposal of depolarized mitochondria [106, 107]. Different mechanisms may regulate mitophagy. The dual system PINK1-Parkin is well described especially in neural tissues. The stabilization of the kinase PINK1 occurs at the surface of mitochondria with low ΔΨ_mito_ with the subsequent recruitment of the ubiquitin ligase Parkin and ubiquitinylation of outer membrane proteins [108]. Mitophagy can be also stimulated by histone deacetylase 6, which is recruited to mitochondria and catalyzes proautophagic cytoplasmic deacetylation reactions [109].

Interestingly, although hepatic PINK1-expression is described [110], to our knowledge, no reports on PINK1 dependent mitophagy in the liver are published. Another mechanism of mitophagy involves the activation of AMPK [111]. AMPK phosphorylates and activates ULK1, one of the initiators of autophagy and the genetic loss of AMPK or ULK1 results in defective mitophagy in mammalian liver and *C. elegans*. These findings showed a conserved mechanism coupling nutrient status with autophagy and cell survival [111]. Interestingly, mitochondrial degradation by autophagy was also described in the liver of GFP-LC3 transgenic mice following nutrient deprivation, reinforcing the results linking AMPK regulation of mitophagy [112].

### 4.3. Mitochondria Can Integrate Energy, Nutrient Metabolism, and Oxidative Stress Responses Determining Cell Fate

Insulin, secreted by pancreatic beta cells upon nutrient stimulation, is one of the most important regulators of nutrient utilization and metabolic homeostasis in the liver. Insulin resistance, a hallmark of NASH and more generally of metabolic syndrome and type II diabetes, is accompanied by reduction of mitochondrial OXPHOS activity and increased ROS production [113]. On the other hand, ROS produced during mitochondrial OXPHOS promote insulin signaling through oxidation of insulin receptor and inhibition of phosphatases, such as PTP1B and PTEN [113]. Importantly, recent investigations pointed out a tight molecular crosstalk between cell survival or cell death pathways and energy metabolism. Using *ex vivo* multinuclear NMR-spectroscopy to study metabolic pathways of [U-(13)C] glucose in mouse liver during Fas-induced apoptosis, Gottschalk et al. found early upregulations in glucose metabolic pathways occurred prior to any visible signs of apoptosis, accompanied by an increased mitochondrial energy production and cellular glutathione synthesis [114]. This metabolic shift seems to potentially contribute to the initiation of apoptosis by mitochondrial energy production and cellular glutathione stores, thus orienting cell fate towards a less pro-inflammatory death. A biochemical analysis using liver mitochondria of two strains of mice (A/J and C57Bl/6, respectively, resistant and susceptible to high-fat diet-induced hepatosteatosis) confirmed a rapid increase by high-fat diet feeding of the respiration rate in A/J but not C57Bl/6 mice. Importantly, ATP production was the same in both types of mitochondria, indicating increased uncoupling of the A/J mitochondria [115]. These results suggest that livers can adapt to high-fat diet feeding by increasing the activity of the oxidative phosphorylation chain and its uncoupling to dissipate the excess of incoming metabolic energy and to reduce the production of ROS [115].

As we mentioned above, liver mitochondria are essential in ammonia detoxification following protein catabolism. In recent years, studies from several laboratories have uncovered a number of factors and pathways that appear to be critically involved in the pathogenesis of hepatic encephalopathy. Foremost is oxidative and nitrosative stress (ONS) and the MMP playing major roles in the mechanism of ammonia-induced astrocyte swelling [116]. The accumulation of intramitochondrial glutamine has been involved. Norenberg et al. [117] were first to describe that the newly synthesized glutamine could be toxic when subsequently metabolized in mitochondria by phosphate-activated glutaminase, yielding glutamate and ammonia. Thus, glutamine can be considered as a carrier of ammonia. The authors propose to consider the intramitochondrial glutamine as a Trojan horse that interferes with mitochondrial function giving rise to excessive production of free radicals and induction of the MPT, two phenomena known to bring about astrocyte dysfunction, including cell swelling.

Moreover, an ammonia-induced increase in intracellular Ca^2+^ has been described which activates a number of enzymes promoting the synthesis of reactive oxygen-nitrogen species, including constitutive nitric oxide synthase, NADPH oxidase and phospholipase A2. ONS subsequently induces the opening of PTP and activates mitogen-activated protein (MAP) kinases and the transcription factor nuclear factor-kappaB (NF-*κ*B). These factors act to generate additional reactive oxygen-nitrogen species, to phosphorylate various proteins and transcription factors, and to cause mitochondrial dysfunction [26]. The pathways and factors described above provide attractive targets for identifying agents potentially useful in the therapy of HE and other hyperammonemic disorders. The most promising of them is the glutamate/glutamine cycle. Indeed, in hyperoxia, glutamine has been described to protect cellular structures, especially mitochondria, from damage. This has been attributed to the activity of the tricarboxylic acid cycle enzyme alpha-ketoglutarate dehydrogenase that was partially protected by its indirect substrate, glutamine, indicating a mechanism of mitochondrial protection [118]. Glutamate dehydrogenase (GDH), a mitochondrial enzyme linking the Krebs cycle to the multifunctional amino acid glutamate could be also an interesting target. Indeed, GDH controls production and consumption of glutamate. GDH activity is under the control of several regulators, conferring to this enzyme energy-sensor property. Indeed, GDH directly depends on the provision of the cofactor NADH/NAD(+), rendering the enzyme sensitive to the redox status of the cell. Moreover, GDH is allosterically regulated by GTP and ADP. GDH is also regulated by ADP-ribosylation, mediated by a member of the energy-sensor family sirtuins, namely, SIRT4. In the brain, GDH ensures the cycling of the neurotransmitter glutamate between neurons and astrocytes. GDH also controls ammonia metabolism and detoxification, mainly in the liver and kidney. Eng and Abraham [119] have described that ammonia, generated from Gln deamination (glutaminolysis) in mitochondria, functions as an autocrine- and/or paracrine-acting stimulator of autophagic flux. Recently, Nissim et al. [120] reported a downregulation of hepatic urea synthesis by oxypurines. Indeed, xanthine and uric acid, both physiologically occurring oxypurines, inhibited the hepatic synthesis of N-acetylglutamate, the key regulator of the first step of mitochondrial urea cycle.

As discussed above, mitochondria are a main source of ROS in hepatocytes and ROS importantly contribute in liver health and disease. While ROS has been commonly associated to lipid, protein, and DNA oxidation and consequent cellular damage, recent studies have shown that mitochondria-generated ROS may be regulated and may regulate many signaling pathways [27, 121]. Oxidative stress may activate prosurvival pathways in hepatocytes, such as NF-*κ*B and NRFs [122–124]. NF-*κ*B regulates a complex network of pathways, as it is known to control the transcription of over 150 genes [122]. Depending on cell type, microenvironmental conditions and eventually costimulated pathways, NF-*κ*B may exert either a pro-survival or a proapoptotic function [122, 125]. In the context of hepatic oxidative stress, it has been shown that NF-*κ*B may induce antiapoptotic factors, such as XIAP, and function like antioxidants in preventing TGF-beta 1-JNK induced-apoptosis [126]. Moreover, NF-*κ*B collaborates with p38 MAP kinase signaling cascade to protect hepatocytes from liver injury induced by TNF-alpha [125]. NRFs also regulates oxidative stress response in the liver. In particular, NRF-1 has been shown to promote cell survival of hepatocytes during development, sustaining the transcription of antioxidant genes and protecting embryonic hepatocytes from TNF-mediated apoptosis [124]. Moreover, NRF-1 has been shown to be induced under prooxidant conditions and to promote the transcription of mitochondrial transcription factor A (Tfam), required for mitochondrial DNA transcription and replication [123]. Hypoxia is another clear example of cell signaling mediated by ROS. Hypoxia is a clinical relevant event both in liver ischemia/reperfusion injury and in hepatocellular carcinoma development. It leads to an increase in production of H_2_O_2_ from mitochondrial complex III, thus creating a cytosolic signal that stabilizes the hypoxia inducible transcription factors HIF-1 [27, 127]. Moreover, during hypoxia ROS activates AMPK, which in turn phosphorylates Na/K ATPase (in order to promote its endocytosis) and mTOR (in order to decrease protein translation), thus contributing to energy conservation [128, 129]. In addition, hypoxia-induced mitochondrial ROS enhance the DNA binding of NF-*κ*B through a redox-dependent mechanism involving the mitochondrial glutathione (mGSH) pool in cancer cells, including hepatoma cell lines [130, 131]. In this context, mGSH regulates the intensity of ROS diffusion in the cytoplasm, allowing activation of the c-Src kinase, with subsequent phosphorylation of the inhibitory subunit I*κ*B, activation of NF-*κ*B and promotion of cancer cell survival [127, 130]. The liver is one of the organs with the highest content of GSH and mGSH plays a central role in regulating both in antioxidant defense against excessive ROS production and in regulation of ROS signaling in liver physiology and pathology [130]. Alcohol consumption has been shown to sensitize hepatocytes to TNF because of mGSH depletion through impaired transport of GSH to mitochondria [132]. Interestingly, GSH transport impairment and TNF sensitization correlate with free cholesterol accumulation in mitochondrial membranes and seem to be a common pro-inflammatory mechanism in both alcoholic and nonalcoholic steatohepatitis [133]. Similar alterations in mGSH regulation have been reported in liver cirrhosis, in particular in an experimental model of secondary biliary cirrhosis in rats induced by bile-duct ligation [134, 135].

During the past decade, a new family of enzymes, the nicotinamide-adenine-dinucleotide- (NAD-) dependent protein deacetylases named sirtuins, has been described to contribute to extended lifespan many animal models, including mammals [136]. Interestingly of the six mammalian sirtuins, three (SIRT3, 4, and 5) are expressed in the mitochondria where they mediate physiologic adaptation to reduced energy consumption [137]. In the liver, SIRT4 activity was shown to decline during calorie restriction, allowing the consumption of glutamine as a fuel source for glucose synthesis. Moreover, SIRT4 depletion increased fatty acid oxidation [138]. Mitochondrial sirtuins could be also interesting targets in the regulation of ammonia production and disposal. Nakagawa et al. [139] have shown that the sirtuin SIRT5 activates CPS1, which we mentioned before as the first enzyme in the urea cycle. In mice, NAD in liver mitochondria increases during fasting, thereby triggering SIRT5-mediated deacetylation of CPS1 and adaptation to increase in amino acid catabolism. These data indicate SIRT5 also has an emerging role in the metabolic adaptation to fasting, high protein diet and calorie restriction. Finally, recent findings correlate SIRT3 to the production of ROS. In particular, SIRT3^−/−^ cells produce increased levels of ROS and have concomitantly a reduced ATP production [11, 12, 140]. These results suggest that SIRT3-mediated deacetylation of electron transport chain may render OXPHOS more efficient [137]. Moreover, SIRT3 may deacetylate and activate the antioxidant enzyme mitochondrial superoxide dismutase (SOD2) and the isocitrate dehydrogenase 2, which generates NADPH for the glutathione synthesis, in mice [141–143]. The sirtuins' antiaging role and their ability of controlling energy metabolism make them interesting target in cancer and metabolic diseases. Interestingly, in a mouse model of metabolic syndrome-associated liver cancer, overexpression of SIRT1 reduced the susceptibility to liver cancer and improved hepatic protection from both DNA damage and metabolic damage [136]. However, recent studies showed that SIRT1 was upregulated in HCC and it has a role in telomere maintenance [144, 145]. Downregulation of SIRT1 suppressed proliferation of HCC cells and induced cellular senescence or apoptosis [144]. Finally, many recent papers show a possible synergic action of cytosolic and mitochondrial sirtuins in regulating glucose and lipid metabolism in the liver [138, 140, 146–148]. SIRT1 has been shown to regulate hepatic glucose and lipid metabolism by activating AMPK and by inducing gluconeogenic genes via activation of PGC-1 alpha in hepatic cell and mouse liver [146, 147]. Interestingly, SIRT1 did not regulate the PGC-1 alpha effects on mitochondrial biogenesis. The mitochondrial SIRT3 was shown to positively modulate fatty acid oxidation and ATP production, in particular deacetylating the long-chain acyl-CoA dehydrogenase and Complex I of the electron transport chain [140, 148]. Finally, in a recent paper, high-fat diet induced a decrease of hepatic SIRT3, hyperacetylation of mitochondrial proteins and fatty liver in mice [149].

Altogether, the studies reviewed show that mitochondria are much more dynamic organelles than considered traditionally. They are key organelles in the integration and adaptation to external stimuli, such as changing composition of diet (i.e., calorie restriction *versus* high fat diet), hypoxia, cold exposure, or physical exercise [150]. Mitochondrial homeostasis is a highly controlled process balancing organelle biogenesis and degradation (essentially by autophagy/mitophagy) and an alteration of this balance may bring to organelle dysfunction, contributing to the development of liver chronic diseases.

## 5. Conclusions

The liver is one of the organs richest in mitochondria. Hepatic mitochondria have unique features compared to other organs' mitochondria, since they are the hub that integrates hepatic metabolism of carbohydrates, lipids, and proteins. Thus, correct functioning of hepatic mitochondria is essential not only to prevent liver disease, such as NAFLD, but also to avoid systemic diseases, such as ammonia-induced hepatic encephalopathy. Mitochondria are also essential in hepatocyte survival as mediator of apoptosis and necrosis. Hepatocyte cell death is involved in most liver pathologies, such as alcoholic and nonalcoholic steatohepatitis, viral hepatitis, liver fibrosis, and carcinogenesis. Hepatocytes have developed different mechanisms to keep mitochondrial integrity or to prevent the effects of mitochondrial lesions, in particular regulating organelle biogenesis and degradation. A better knowledge of the mechanisms and pathways involved in mitochondria homeostasis should improve preventive and therapeutic strategies for liver diseases.

## Abbreviations

2-AAF: 2-Acetylaminofluorene
DAG: Diacylglycerol
GS: Glutamine synthetase
GSH: Glutathione
HCC: Hepatocellular carcinoma
MIM: Mitochondrial inner membrane
MMP: Mitochondrial membrane permeabilization
MOM: Mitochondrial outer membrane
MRC: Mitochondrial respiratory chain
NAG: N-acetyl-L-glutamate
NASH: Nonalcoholic steatohepatitis
OXPHOS: Oxidative phosphorylation
PTP: Permeability transition pore
ROS: Reactive oxygen species.
